# Drug efficacy by direct and adjusted indirect comparison to placebo: An illustration by *Mycobacterium avium *complex prophylaxis in HIV

**DOI:** 10.1186/1742-6405-8-14

**Published:** 2011-03-10

**Authors:** Jennifer Chu, Caroline E Sloan, Kenneth A Freedberg, Yazdan Yazdanpanah, Elena Losina

**Affiliations:** 1 Division of General Medicine, Department of Medicine, Massachusetts General Hospital, Boston, USA; 2 Division of Infectious Disease, Department of Medicine, Massachusetts General Hospital, Boston, USA; 3 The Harvard University Center for AIDS Research, Harvard Medical School, Boston, USA; 4 Department of Orthopaedic Surgery, Brigham and Women's Hospital, Boston, USA; 5 Faculté de Médecine de Lille, Centre Hospitalier de Tourcoing, Tourcoing, France

## Abstract

**Background:**

Our goal was to illustrate a method for making indirect treatment comparisons in the absence of head-to-head trials, by portraying the derivation of published efficacies for prophylaxis regimens of HIV-related opportunistic infections.

**Results:**

We identified published results of randomized controlled trials from the United States in which HIV-infected patients received rifabutin, azithromycin, clarithromycin, or placebo for prophylaxis against *Mycobacterium avium *complex (MAC). We extracted the number of subjects, follow-up time, primary MAC events, mean CD4 count, and proportion of subjects on mono or dual antiretroviral therapy (ART) from each study. We derived the efficacy of each drug using adjusted indirect comparisons and, when possible, by direct comparisons. Five articles satisfied our inclusion criteria. Using direct comparison, we estimated the efficacies of rifabutin, clarithromycin, and azithromycin compared to placebo to be 53% (95% CI, 48-61%), 66% (95% CI, 61-74%), and 66% (95% CI, 60-81%), respectively. Using adjusted indirect calculations, the efficacy of rifabutin compared to placebo ranged from 41% to 44%. The adjusted indirect efficacies of clarithromycin and azithromycin were estimated to be 73% and 72%, respectively.

**Conclusions:**

Accurate estimates of specific drug dosages as compared to placebo are important for policy and implementation research. This study illustrates a simple method of adjusting for differences in study populations by using indirect comparisons in the absence of head-to-head HIV clinical trials.

## Background

Cost-effectiveness analyses are frequently used to guide health policy decisions, particularly in HIV disease[1-3]. To offer long term projections on clinical and economic implications to specific treatment strategies and to address the need to make clinical decisions where evidence from published studies is insufficient, cost-effectiveness analyses offer strategic insights using model-based evaluations. Models used in cost-effectiveness analyses are often multidimensional and based on a large number of input parameters. In such model-based evaluations, efficacy estimates of drug regimens compared to placebo are critical for accurate delineation of alternative treatment strategies and cost-effectiveness comparisons. However, head-to-head placebo-controlled trials often are not feasible; they are expensive, time-consuming, and unethical if guidelines for a pharmaceutical intervention already exist [4]. Adjusted indirect comparison of randomized controlled trials has become an increasingly accepted method for assessing the effect of pharmaceutical interventions on survival outcomes, in the absence of placebo-controlled trials [5-8]. Within the framework of a cost-effectiveness model, often based on hundreds of parameters, it is not always feasible to use complex methods to derive every input parameter, especially for parameters not likely to affect major policy decisions.

Our goal was to illustrate a simple method for adjusting drug efficacy estimates according to differences in disease severity to derive parameters for a complex computer simulation model of HIV disease[1,9]. One study, for example, may compare regimen A to regimen B, and another study may compare regimen B to placebo. Adjusted indirect comparison provides a method for establishing the efficacy of regimen A compared to placebo, without losing the positive attributes of randomization.

Previous studies using adjusted indirect comparison estimated one-time probabilities and pooled the efficacies of drug regimens with varying doses [5,7,10,11]. Here, we establish a method for determining the efficacy of specific drug doses over time, thus allowing for predictions of treatment failure after any duration of therapy. We focused our illustration on prophylactic drugs for *Mycobacterium avium *complex (MAC) in patients infected with the human immunodeficiency virus (HIV) in the United States, because national guidelines recommend administering specific drugs and doses to prevent MAC [12]. Moreover, we also selected MAC as our illustration because of the availability of placebo-controlled trials for each guideline-recommended drug.

## Methods

### Study selection

We performed a MEDLINE search to identify randomized controlled trials of primary prophylaxis against MAC that were consistent with the current United States prophylaxis guidelines for HIV-infected patients[12]. We used the following search terms: *Mycobacterium avium *complex, randomized-controlled trial, placebo, rifabutin, azithromycin, and clarithromycin. We then reviewed the bibliographies of selected articles to identify other relevant studies. We considered data from randomized controlled clinical trials that reported follow-up time and administered primary prophylaxis for MAC, using one of the following drug regimens: 300 mg rifabutin once daily, 1200 mg azithromycin once weekly, or 500 mg clarithromycin twice daily. These doses are based on the 2009 "Guidelines for Prevention and Treatment of Opportunistic Infections in HIV-infected Adults and Adolescents" [12]. To be included in this analysis, studies had to have at least two treatment arms and compare prophylactic regimens either to placebo directly, or to one another. Data on the number of subjects, follow-up time, and primary MAC events are included in Table 1. We collected additional data on mean CD4 count, number of patients on mono or dual antiretroviral therapy (ART), endpoint definitions, and inclusion or exclusion criteria from each study. For one pair of identically designed studies, we derived efficacy using the weighted averages of data from the two studies [13].

**Table 1:** Characteristics of 5 randomized controlled trials of primary prophylaxis against *Mycobacterium avium *complex in HIV-infected adults

Study	Drug dose	No. subjects	Mean CD4 Count ^a ^(cells/μl)	% On ART	Median Follow-up time ^b ^(days)	Primary MAC events (N)	Direct monthly failure rate (95% CI)	Direct monthly probability of failure
*Nightingale 1993, study 023 and 027 *^c ^[13]	Rifabutin, 300 mg, qd	283	64	100	209 ^d^	24	0.012 (0.007-0.017)	0.012
	Placebo	290	56	100	202 ^d^	51	0.027 (0.019-0.0034)	0.026
*Havlir 1996 *[17]	Rifabutin, 300 mg, qd	223	47	--	514	52	0.014 (0.010-0.018)	0.014
	Azithromycin, 1200 mg, qwk	223	49	--	514	31	0.008 (0.005-0.011)	0.008
*Benson 2000 *[16]	Rifabutin, 300 ^e ^mg, qd	391	30	75	574	59	0.008 (0.006-0.010)	0.008
	Clarithromycin, 500 mg, bid	398	27	73	595	36	0.005 (0.003-0.006)	0.005
*Pierce 1996 *[14]	Clarithromycin, 500 mg, bid	333	30	--	427 ^f^	19	0.004 (0.002-0.006)	0.004
	Placebo	334	25	--	402 ^f^	53	0.012 (0.009-0.015)	0.012
*Oldfield 1998 *[15]	Azithromycin, 1200 mg, qwk	85	44	--	400 ^d^	9 ^g^	0.008 (0.003-0.013)	0.008
	Placebo	89	44	--	340 ^d^	24 ^g^	0.024 (0.015-0.034)	0.024

qd: once a day; bid: twice a day; qwk: once a week; MAC: *Mycobacterium avium *complex; ART: antiretroviral therapy; CI: confidence interval

^a ^At baseline

^b ^All patients on ART were on dual or mono therapy

^c ^Study 023 and 027 are two identically designed studies. We calculated weighted averages the number of subjects, follow-up time, and number of new MAC events for the two studies.

^d ^Duration on treatment

^e ^This study was originally designed with a 450 mg qd dosage but reduced to 300 mg qd after 9 months.

^f ^Mean follow-up time

^g ^The primary endpoints of this study were MAC symptoms and positive culture. We only included culture-positive events, to remain consistent with the other studies, which all used positive MAC cultures as primary endpoints.

### Direct comparison

If Trial 1 compared regimen A to placebo, we used Equation 1 to derive the efficacy of regimen A relative to placebo.(1)

We determined the two-sided 95% confidence interval (CI) of each efficacy derived by direct comparison.

### Adjusted indirect comparison

When direct comparison of a drug regimen to placebo was not possible, we made adjusted indirect comparisons. For example, when trial 1 compared regimen A to placebo, and trial 2 compared regimen B to regimen A, we computed a "correction" factor to adjust for differences in baseline characteristic differences, including mean CD4 count and number of patients on ART, between the subjects of trial 1 and trial 2. The correction factor preserved the balance between the two randomized groups. Using Equation 2, we derived a correction factor to compare regimen A of trial 2 to regimen A of trial 1.(2)

We then used Equation 3 to calculate the adjusted monthly probability of failure of regimen B.(3)

This adjusted monthly probability of failure allowed us to compare regimen B in trial 2 to placebo in trial 1. We obtained the efficacy of regimen B using Equation 4.(4)

We compared the direct and adjusted indirect efficacies of each regimen to assess the validity of adjusted indirect comparisons.

## Results

### Characteristics of eligible trials

We identified five eligible randomized controlled trials that included a total of 3,222 subjects (Table 1). Three studies compared one drug regimen to placebo, and two trials compared different prophylaxis regimens to each other.

### Efficacy by direct comparison

We used data from three studies, by Nightingale *et al*., Pierce *et al*., and Oldfield *et al*. to compare rifabutin, clarithromycin, and azithromycin to placebo, directly (Table 2) [13-15]. The absolute efficacies of rifabutin, clarithromycin, and azithromycin, each compared to placebo, were estimated to be 53% (95% CI, 48-61%), 66% (95% CI, 61-74%), and 66% (95% CI, 60-81%).

**Table 2:** Efficacy of MAC regimens by direct and adjusted indirect comparison

Drug dose and study	Method of efficacy derivation	Study used for comparison	Correction factor	Adjusted monthly probability of failure	% Efficacy (95% CI)
**Rifabutin, 300 mg, qd**					
*Nightingale 1993 *[13]	Direct	--	--	--	53 (48-61)
*Havlir 1996 *[17]	Adjusted indirect	*Oldfield 1998*	0.979 ^a^	0.014	44
*Benson 2000 *[16]	Adjusted indirect	*Pierce 1996*	0.879 ^b^	0.007	41
**Clarithromycin, 500 mg, bid**					
*Pierce 1996 *[14]	Direct	--	--	--	66 (61-74)
*Benson 2000 *[16]	Adjusted indirect	*Nightingale 1993*	1.542 ^c^	0.007	73
**Azithromycin, 1200 mg, qwk**					
*Oldfield 1998 *[15]	Direct	--	--	--	66 (60-81)
*Havlir 1996 *[17]	Adjusted indirect	*Nightingale 1993*	0.896 ^d^	0.007	72

Comparison of the monthly failure probabilities of:

^a ^Compared to azithromycin in *Oldfield *1998 [15].

^b ^Compared to clarithromycin in *Pierce *1996 [14]

^c ^Compared to rifabutin in *Nightingale *1993 [13]

^d ^Compared to rifabutin in *Nightingale *1993[13]

### Efficacy by indirect comparison

After adjusting the failure rate of rifabutin in the Benson *et al*. study to baseline characteristics in the Pierce *et al*. study [14,16], we estimated the adjusted indirect efficacy of rifabutin in Benson *et al*. to be 41%. Similarly, we compared rifabutin in Havlir *et al*. to placebo in Oldfield *et al*., because both studies contained one azithromycin arm [15,17]. The efficacy of rifabutin in Havlir *et al*. was 44%, compared to placebo in Oldfield *et al*.

When we adjusted the results of the Benson *et al*. study to baseline characteristics in Nightingale *et al*., using the rifabutin arms in each study, we estimated the efficacy of clarithromycin in Benson *et al*. compared to placebo in Nightingale *et al*. to be 73% [13,16]. When we adjusted the results of the Havlir *et al*. study to baseline characteristics in Nightingale *et al*. using the rifabutin arms in each study [13,17], we estimated the efficacy of azithromycin in the Havlir *et al*. to be 72%, compared to the Nightingale *et al*. placebo arm.

### Comparison of direct and adjusted indirect comparison methods

The efficacies of clarithromycin and azithromycin derived by adjusted indirect comparison were not significantly different from the efficacies derived by direct comparison. However, our estimate of the efficacy of rifabutin by indirect comparison (41-44%) was significantly lower than the efficacy derived by direct calculation (53%).

## Discussion

This paper illustrates a simple method that can be used to estimate input values for auxiliary parameters in multidimensional cost-effectiveness models. Since thorough methodological expertise in indirect comparisons may not always be accessible, the method illustrated in this paper could be used to derive efficacy of treatments where direct trials based on data are not readily available. To establish the efficacy of a drug regimen, it is necessary to compare outcomes for patients on and off therapy. While it is sometimes possible to derive this information directly from the results of randomized controlled trials, clinical trials are expected to provide enrolled participants with the best proven treatment, or at least the standard of care [4]. Thus, most studies compare different treatment options; studies that administer placebo to some subjects despite existing and accepted treatment options for the disease of interest lack equipoise and therefore are not ethical or feasible [4]. In this paper we have illustrated a simple method for indirectly estimating the dose-specific efficacy of drug regimens from reported results of randomized controlled trials without placebo arms by a straightforward adjustment for baseline clinical severity. When possible, we estimated the efficacies directly from the trials.

We found that the derived adjusted indirect efficacies of clarithromycin and azithromycin were similar to corresponding direct efficacies. However, the indirect efficacy of rifabutin was significantly lower than the efficacy derived by direct comparison. Unlike most other studies used in this analysis, the Nightingale *et al*. study reported mean duration on treatment, which is shorter than mean follow-up time. This substitution may therefore have led to an overestimation of the direct efficacy of rifabutin. The greater efficacy of rifabutin in the direct comparison may also be attributed to the greater proportion of patients on ART in this trial.

Our proposed method was consistent with that of previous studies showing that adjusted indirect comparison reduces bias in drug efficacy calculations [5-7,11]. Our inclusion criteria were stricter than those in previous studies, because we examined outcomes only from trials that compared drug regimens with specific doses and that provided results at several time points. Thus, we avoided having to pool results from various doses of the same drug regimen. Our results may be more accurate than previous studies for the specific doses examined, since we only included trials that administered the doses recommended in the United States "Guidelines for Prevention and Treatment of Opportunistic Infections in HIV-Infected Adults and Adolescents"[12]. Similar results may be obtained using Indirect Treatment Comparisons (ITC) Software from the Canadian Agency for Drugs and Health Technologies [18]. While this offers a means of validation of the methods in this paper, a step-by-step description may be useful to those who do not have direct access to the ITC software, or for further understanding of the insights provided.

One of the main purposes of the indirect comparisons is to make stronger inferences about comparisons being studied. We recognize the scarcity of placebo-controlled trials in the HIV/AIDS field, particularly among newer trials, and we believe that using older placebo-controlled trials, as we have done in our illustration, for the purpose of adjusted indirect comparisons, is acceptable. Our study was limited by the number of studies that could be used to derive OI prophylaxis efficacy. Only five studies met the inclusion criteria. However, because the focus of this analysis was to illustrate simple and replicable methodology for adjusted indirect comparison of drug regimens, the small number of included studies does not deter from this goal. Moreover, two studies did not report follow-up time [13,15]. For these studies, we calculated efficacy by substituting follow-up time with mean duration on treatment to calculate efficacy. The Oldfield *et al*. study was terminated early because administering placebo became inappropriate when the results of a separate azithromycin efficacy trial [15]. It may be reasonable to assume that most patients were on treatment at the time of study discontinuation, and thus that the unreported mean follow-up time is very similar to the mean duration on treatment. However, treatment duration in the Nightingale *et al*. study may have been greater than the true unreported mean follow-up time, and could have led to an overestimation of the efficacy of rifabutin. While this method offers a useful approach for derivation of point estimates, an extensive set of sensitivity analyses are necessary to examine the robustness of policy conclusions to uncertainty in parameter values. If a parameter is influential, more sophisticated methods should be employed to obtain a more precise value of parameter.

The prevalence of MAC and other opportunistic infections among HIV-infected patients in the United States and Europe has greatly decreased since the earlier years of the HIV epidemic, due to the success of combination antiretroviral therapy [19]. However, methods presented in this study continue to be applicable to resource-limited settings, where the use of opportunistic infection prophylaxis in the absence of ART is still widespread. In these areas, the WHO recommends lifelong prophylaxis for fungal and bacterial infections, as well as for *Pneumocystis carinii *Pneumonia with drugs such as fluconazole and cotrimoxazole [20]. In the United States, recommendations for the prevention of opportunistic infections continue to be revised regularly in the national guidelines [12,21,22]. Similar indirect comparison methods may be useful in comparing effective first-line antiretroviral regimens in the United States and in many countries–such as those containing efavirenz, darunavir, atazanavir, and raltegravir in the United States–that have not been compared directly with each other [23-25]. These methods can also be used to compare second-line or subsequent ART regimens when efficacy data have been published but direct comparisons may have not been done.

## Conclusion

The methodology demonstrated in this study is applicable to policy and implementation research, for which it is necessary to know the absolute efficacy of specific doses of pharmaceutical interventions as compared to no intervention, to predict the outcomes of treatment policies. As treatment options for HIV disease, both in terms of opportunistic infection prophylaxis and ART, continue to grow, these methods can help estimate efficacies across a wide range of available and useful therapeutic regimens.

## Competing interests

The authors declare that they have no competing interests.

## Authors' contributions

JC, CS, and EL conceived and designed the study. JC and CS drafted the manuscript. KF, YY, and EL provided critical revisions of the article for important intellectual content. All authors read and approved the final manuscript.
